# Outcomes among patients with chronic obstructive pulmonary disease after recovery from COVID-19 infection of different severity

**DOI:** 10.1038/s41598-024-64670-9

**Published:** 2024-06-16

**Authors:** Wang Chun Kwok, Chi Hung Chau, Terence Chi Chun Tam, Fai Man Lam, James Chung Man Ho

**Affiliations:** 1 Department of Medicine, Queen Mary Hospital, The University of Hong Kong, 4/F, Professorial Block, 102 Pokfulam Road, Hong Kong, Hong Kong Special Administrative Region People’s Republic of China; 2 Tuberculosis and Chest Unit, Grantham Hospital, Hong Kong, Hong Kong Special Administrative Region People’s Republic of China

**Keywords:** COVID-19, COPD, COPD control, COPD exacerbation, Microbiology, Health care, Medical research

## Abstract

While studies have suggested increased risks of severe COVID-19 infection in chronic obstructive pulmonary disease (COPD), the persistent and delayed consequences of COVID-19 infection on patients with COPD upon recovery remain unknown. A prospective clinical study was conducted in Hong Kong to investigate the persistent and delayed outcomes of patients with COPD who had COVID-19 infection of different severity (mild-moderate COVID-19 and severe COVID-19), compared with those who did not. Chinese patients with COPD ≥ 40 years old were recruited from March to September 2021. They were prospectively followed up for 24.9 ± 5.0 months until 31st August 2023. The primary outcome was the deterioration in COPD control defined as the change in mMRC dyspnea scale. The secondary outcomes included the change in exacerbation frequency and non-COVID-19 respiratory mortality (including death from COPD exacerbation or bacterial pneumonia). 328 patients were included in the analysis. Patients with mild-moderate and severe COVID-19 infection had statistically significant increased risks of worsening of mMRC dyspnoea scale by increase in 1 score from baseline to follow-up with adjusted odds ratios of 4.44 (95% CI = 1.95–10.15, *p* < 0.001) and 6.77 (95% CI = 2.08–22.00, *p* = 0.001) respectively. Patients with severe COVID-19 infection had significantly increased risks of increase in severe COPD exacerbation frequency with adjusted odds ratios of 4.73 (95% CI = 1.55–14.41, *p* = 0.006) non-COVID-19 respiratory mortality from COPD exacerbation or pneumonia with adjusted hazard ratio of 11.25 (95% CI = 2.98–42.45, *p* < 0.001). After recovery from COVID-19, worsening of COPD control from worsening of dyspnea, increase in severe exacerbation frequency to non-COVID-19 respiratory mortality (COPD exacerbation and pneumonia) was observed among patients with severe COVID-19. Mild to moderate COVID-19 was also associated with symptomatic deterioration.

After the coronavirus disease 2019 (COVID-19) pandemic, there has been a major focus on the impact of sustained and delayed impact of COVID-19 on various chronic diseases^1–3^. There were reports suggesting that physical and psychological symptoms could occur following COVID-19, including fatigue, dyspnea, cardiac abnormalities, cognitive impairment, sleep disturbances, symptoms of post-traumatic stress disorder, muscle pain, concentration problems, and headache^1,4,5^. The organ systems involved by long COVID may include the respiratory, cardiovascular, neurological, gastrointestinal, and musculoskeletal systems^6^. Poor pre-pandemic general health and underlying asthma were shown to be associated with long COVID^7^. It has also been suggested that mild-to-moderate COVID-19 among asthma patients, upon recovery, was associated with worsening of asthma symptom, lower asthma control test (ACT) score, a higher need for escalation of asthma maintenance therapy and more uncontrolled asthma^8^. For chronic obstructive pulmonary disease (COPD), some studies suggested that it was associated with increased risks of developing long COVID symptoms^9,10^. Patients with COPD also had increased risks of mortality when they had COVID-19^11^. The evidence on COVID-19 and COPD mainly focused on the severity of COVID-19 and post-COVID syndrome. There has been a lack of evidence on COVID-19 on COPD control, including symptoms, exacerbation rate and mortality, in the medium- to long-term.

We hereby conducted this prospective study to assess the association between COVID-19 of different severity and COPD control upon recovery from acute infection with detailed and objective assessment of COPD control using various clinical parameters.

## Methods

### Study design and data sources

Chinese patients ≥ 40 years old who had COPD followed up at Queen Mary Hospital (QMH) and Grantham Hospital (GH) in Hong Kong were prospectively recruited in year 2021. This was a cohort of COPD patients which was followed up prospectively for a duration of 24.9 ± 5.0 months from March to September 2021 and continued till 31/08/2023. The diagnosis of COPD was confirmed by spirometry demonstrating post-bronchodilator airflow limitation with forced expiratory volume in one second/forced vital capacity [FEV_1_/FVC] ratio < 0.7. Exclusion criteria comprised of co-existing asthma, bronchiectasis, interstitial lung disease and COVID-19 before enrollment. Written informed consent was obtained. The recruited patients had history taking, physical examination and blood taking for complete blood count at the time of recruitment. The relevant medical records including demographic data, clinical data / investigations, and use of ICS/bronchodilators including long-acting beta-agonists (LABA) and long-acting muscarinic antagonists (LAMA) were all recorded in the first visit. Regular use of ICS, LABA and LAMA was defined as continuous use for at least 12 months prior to enrollment. Those who had COVID-19 were categorized into mild-moderate and severe COVID-19 groups. Patients who did not have COVID-19 throughout the entire study period were classified as non-COVID-19 group. Mild disease was defined as having any of the various signs and symptoms of COVID-19 (e.g., fever, cough, sore throat, malaise, headache, muscle pain, nausea, vomiting, diarrhea, loss of taste and smell) but who do not have shortness of breath, dyspnea, or abnormal chest imaging. Moderate disease was defined as having evidence of lower respiratory disease during clinical assessment or imaging, with oxygen saturation measured by pulse oximetry ≥ 94% on room air. Severe disease was defined as having SpO_2_ < 94% on room air, ratio of arterial partial pressure of oxygen to fraction of inspired oxygen (PaO_2_/FiO_2_) < 300 mm Hg, respiratory rate > 30 breaths/min, or lung infiltrates > 50%^12^. Patients who died of COVID-19 and lost to follow-up were excluded. The diagnosis of COVID-19 was laboratory-confirmed by positive reverse transcription–polymerase chain reaction (RT-PCR) test, or positive rapid antigen test (RAT), as documented on the designated COVID-19 data platform on Clinical Management System (CMS) of Hong Kong Hospital Authority (HA). Patients’ records were accessed through the electronic patient record (ePR) of HA, which consisted of records of all patients with out-patient clinic attendance and hospital admission. The information available included demographics, clinical notes, investigation results and treatment records.

At the recruitment visit in 2021, demographics (age, gender), clinical data (mMRC (Modified Medical Research Council) dyspnea scale, COPD medications, COPD exacerbation frequency, comorbidities), and spirometry results were recorded. During follow-up, the following clinical data were collected: details of COVID-19 infection (date of infection, hospitalization, and complications), date and dose of COVID-19 vaccination, type of COVID-19 vaccines, mMRC dyspnea scale as well as COPD exacerbation frequency. The patients were followed up till 31/8/2023. COPD exacerbation was defined as an acute event characterized by worsening of respiratory symptoms beyond the normal day-to-day variations, leading to a change in medications. Compatible symptomatology comprised of one or more of the following: (1) increased frequency and severity of cough; (2) increased sputum volume and/or purulence (3) increased dyspnea requiring medical attention and treatment^13^. Mild COPD exacerbation was defined as COPD exacerbation that was treated with short acting bronchodilators only. Moderate COPD exacerbation was defined as COPD exacerbation that was treated with short acting bronchodilators and oral corticosteroid. Severe COPD exacerbation was defined as COPD exacerbation that was treated required hospitalizations or emergency department visits^14^. The mMRC dyspnoea scale were assessed in the recruitment visit and every follow-up visit scheduled at every 12 to 26 weeks interval. Spirometry was performed with CareFusion Vmax® Encore 22 system, with the updated spirometric reference values for adult Chinese in Hong Kong^15^. The study was approved by the Institutional Review Board of the University of Hong Kong and Hospital Authority Hong Kong West Cluster (UW 21–172).

### Outcomes

The primary outcome was the change in COPD control defined by mMRC dyspnea scale in the three patient groups. The secondary outcomes included the change in exacerbation frequency and non-COVID-19 respiratory mortality (including death from COPD exacerbation or bacterial pneumonia) among the three patient groups.

### Statistical analysis

The demographic and clinical data were described in actual frequency, mean ± standard deviation (SD) or median (interquartile range [IQR]). Baseline demographic and clinical data were compared between the patients with or without COVID-19; as well as among patients without COVID-19, mild- moderate COVID-19 and severe COVID-19 by Chi-squared test or Fisher’s exact test as appropriate. Continuous variables were expressed as mean ±SD and compared among the three groups using one-way ANOVA. The risks of worsening COPD control between patients in the three groups were compared by binary logistic regression. Multiple logistic regression modeling was used to account for potential confounders including age, gender, baseline FEV_1_ (% predicted), body mass index (BMI), mMRC dyspnea scale at baseline, annual COPD exacerbation frequency at baseline, COVID-19 vaccination status, baseline blood eosinophil count, COPD medication use at baseline (LABA, LAMA and ICS) and other factors that were significantly different at baseline. Cox-regression was used to estimate the survival. Kaplan–Meier method and the stratified log-rank statistic were used to assess the non-COVID-19 respiratory mortality of the patient groups with respect to the composite primary end point. The statistical significance was determined at the level of *p* < 0.05. All the statistical analyses were performed using the 28th version of SPSS statistical package.

### Ethics approval and consent to participate

The study was approved by the Institutional Review Board of the University of Hong Kong and Hospital Authority Hong Kong West Cluster (reference number: UW 21–172). Informed consent was obtained from all patients. The study was conducted in accordance with the Declaration of Helsinki.

## Results

**Figure 1:**
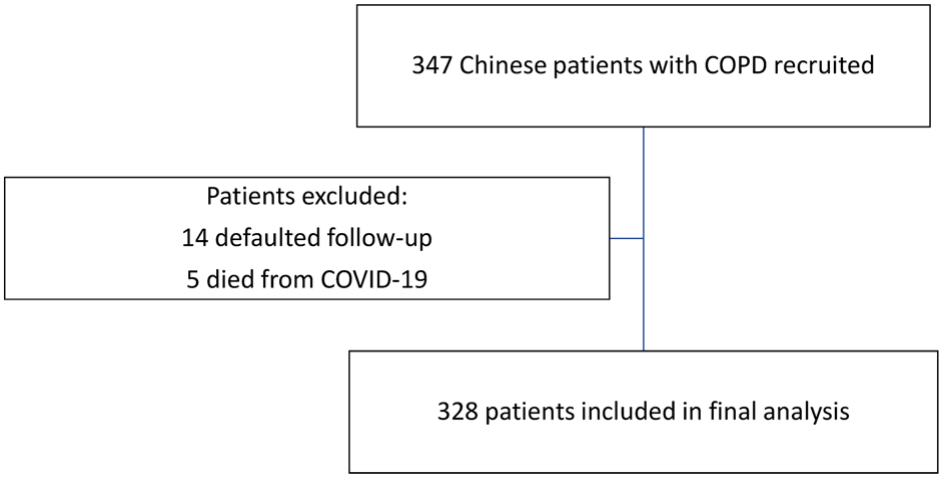
Flow diaphragm on patient selection.

A total of 347 adult patients with COPD were recruited, in which 14 defaulted follow-up and 5 died from COVID-19 infection. The final analysis was performed in the remaining 328 patients. The patient disposition is summarized in Fig. 1.

Among the whole cohort of 328 patients included in the analysis, there were 132 patients (40.2%) having mild-moderate COVID-19 and 37 (11.3%) had severe COVID-19. The mean age was 74.5 ± 8.8 years, predominantly male (90.5%) and mean BMI was 23.1 ± 4.3 kg/m^2^. The mean baseline FEV_1_ was 1.41 ± 0.57 L (63.2 ± 22.8% predicted), with the baseline FEV_1_/FVC ratio was 50.5 ± 15.8%. The median [IQR] mMRC dyspnea scale at baseline assessment was 1 [2, 3]. 44 patients died during a mean follow-up duration of 742 ± 151 days. Among those with COVID-19, the mean duration of follow-up after recovery from COVID-19 was 760 ± 130 days.

The baseline demographics are shown in Table 1 and Supplementary Table 1.

**Table 1:** Baseline demographic and clinical characteristics of COPD patients according to COVID-19 infection status.

	Non-COVID-19 (n = 159)	Mild-to-moderate COVID-19 (n = 132)	Severe COVID-19 (n = 37)	Whole cohort (n = 328)	p-values^
Age (years), mean ± SD	75.8 ± 9.5	72.0 ± 7.7	77.8 ± 7.5	74.5 ± 8.8	< 0.001*
Male gender	139 (87.4%)	125 (94.7%)	33 (89.9%)	297 (90.5%)	0.10
Body mass index (kg/m^2^), mean ± SD	22.8 ± 4.2	23.5 ± 4.1	23.1 ± 5.3	23.1 ± 4.3	0.45
COPD stage by lung function parameters					0.17
Stage 1	37 (23.3%)	30 (22.7%)	6 (16.2%)	73 (22.3%)	
Stage 2	69 (43.4%)	69 (52.3%)	18 (48.6%)	156 (47.6%)	
Stage 3	40 (25.2%)	27 (20.5%)	9 (24.3%)	76 (23.2%)	
Stage 4	8 (8.2%)	6 (4.5%)	4 (10.8%)	18 (5.5%)	
COPD group by GOLD recommendation					0.06
Group A	50 (31.4%)	53 (40.2%)	6 (16.2%)	109 (33.2%)	
Group B	81 (50.9%)	55 (41.7%)	25 (67.6%)	161 (49.1%)	
Group E	28 (17.6%)	24 (18.2%)	6 (16.2%)	58 (17.7%)	
mMRC dyspnoea scale at baseline, mean ± SD	1.66 ± 0.89	1.61 ± 0.88	1.68 ± 0.97	1.66 ± 0.90	
Annual COPD exacerbation at baseline (number per year), mean ± SD	0.25 ± 0.73	0.30 ± 0.91	1.0 ± 1.1	0.35 ± 0.88	< 0.001*
Baseline FEV_1_ (L), mean ± SD	1.37 ± 0.55	1.53 ± 0.61	1.19 ± 0.44	1.41 ± 0.57	0.002*
Baseline FEV_1_ (% predicted), mean ± SD	62.2 ± 22.1	66.2 ± 22.5	56.9 ± 25.7	63.2 ± 22.8	0.08
Baseline FVC (L), mean ± SD	2.75 ± 0.85	3.07 ± 0.85	2.42 ± 0.70	2.84 ± 0.86	< 0.001*
Baseline FVC (% predicted), mean ± SD	92.4 ± 25.2	97.1 ± 23.4	87.6 ± 24.5	93.8 ± 24.5	0.10
Baseline FEV_1_ to FVC ratio, mean ± SD	50.6 ± 13.8	50.7 ± 18.6	49.3 ± 13.3	50.5 ± 15.8	0.88
Baseline eosinophil count (x cells/µL), mean ± SD	230 ± 222	271 ± 208	179 ± 138	241 ± 211	0.04*
Medication					
LABA	121 (76.1%)	96 (72.7%)	24 (64.9%)	241 (73.5%)	0.37
LAMA	125 (78.6%)	105 (79.5%)	28 (75.7%)	258 (78.7%)	0.88
ICS	73 (45.9%)	66 (50.0%)	22 (59.5%)	161 (49.1%)	0.32
Long term oxygen therapy	7 (4.4%)	4 (3.0%)	3 (8.1%)	14 (4.3%)	0.40
Co-morbidities					
Hypertension	18 (11.3%)	24 (18.2%)	9 (24.3%)	51 (15.5%)	0.081
Diabetes mellitus	66 (41.5%)	55 (41.7%)	14 (37.8%)	135 (41.2%)	0.91
Ischaemic heart disease	23 (14.5%)	22 (16.7%)	4 (10.8%)	49 (14.9%)	0.66
History of stroke	2 (1.3%)	4 (3.0%)	0 (0%)	6 (1.8%)	0.36
History of malignancies	28 (17.6%)	22 (16.7%)	2 (5.4%)	52 (15.9%)	0.18

^Between Non-COVID-19, mild-moderate COVID-19 and severe COVID-19 subgroups.

*: statistically significant; SD: standard deviation; GOLD: Global Initiative for Chronic Obstructive Lung Disease; µL: microliters; L: Liter; FEV_1_: forced expiratory volume in one second; FVC: forced vital capacity.

### Change in mMRC dyspnea scale from baseline to the last follow-up

The mean mMRC scores at baseline assessment were 1.66 ± 0.89 in the non-COVID group, 1.61 ± 0.88 in the mild-moderate COVID-19 group and 1.68 ± 0.97 in the severe COVID-19 group. The mean mMRC scores on the last follow-up were 1.80 ± 0.95 in the non-COVID-19 group, 1.88 ± 0.88 in the mild-moderate COVID-19 group and 2.20 ± 0.96 in the severe COVID-19 group.

**Figure 2:**
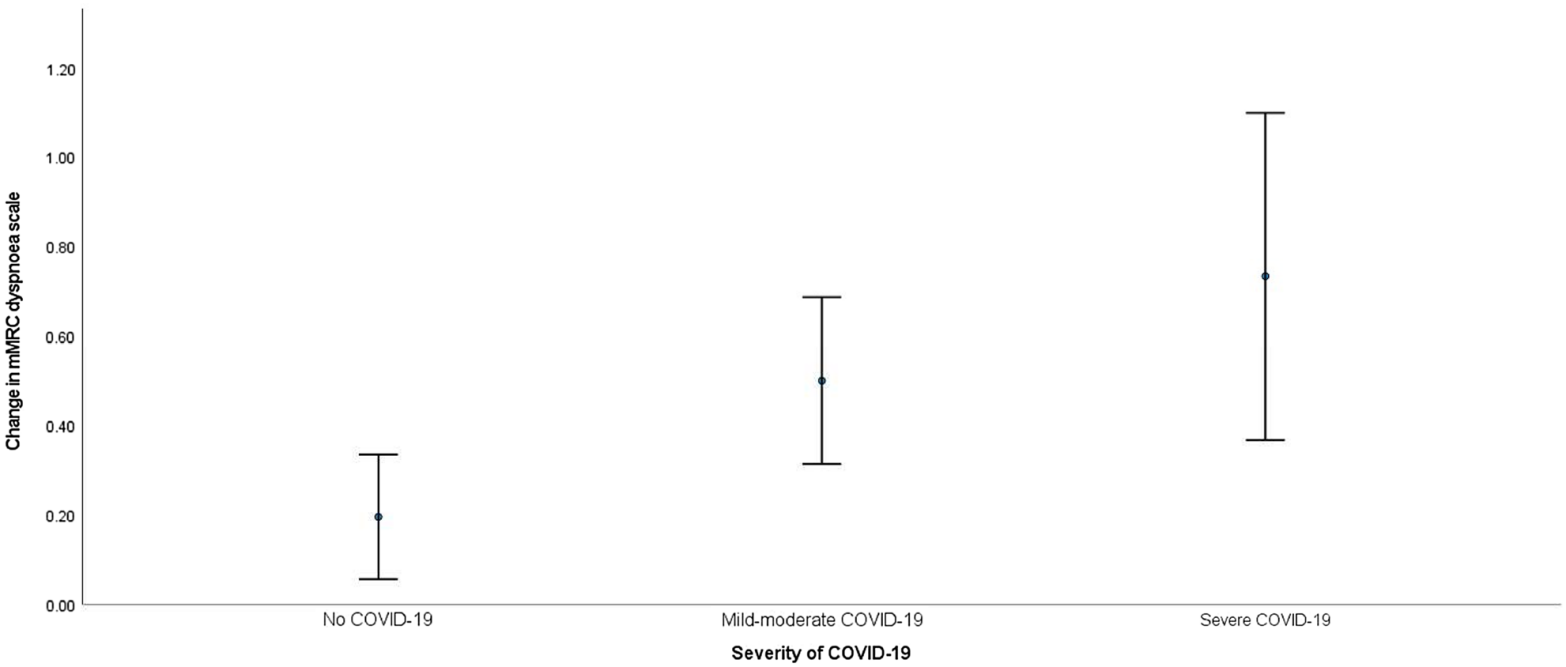
Mean change in mMRC dyspnoea scale among patients without COVID-19, mild-moderate COVID-19 and severe COVID-19, from baseline to follow-up.

The mean change of mMRC dyspnea scores were +0.10 ± 0.41 in the non-COVID group, +0.25 ± 0.53 in the mild-moderate COVID-19 group and + 0.37 ± 0.49 in the severe COVID group, *p* = 0.004 in univariate analysis and 0.002 in multivariate analysis (Fig. 2). 14 (8.8%), 38 (28.8%) and 11 (29.7%) of the patients in the non-COVID-19, mild-moderate COVID-19 and severe COVID-19 groups had worsening of mMRC dyspnoea scale by increase in 1 score from baseline to the last follow-up. The odds ratios (OR) for increase in mMRC dyspnoea scale, using the non-COVID-19 group for comparison, was 3.59 (95% confidence interval [CI] = 1.83–7.02, *p* < 0.001) for the mild-moderate COVID-19 group and 4.92 (95% CI = 1.95–12.34, *p* < 0.001) for the severe COVID-19 group, with the adjusted OR (aOR) 4.44 (95% CI = 1.95–10.15, *p* < 0.001) and 6.77 (95% CI = 2.08–22.00, *p* = 0.001) respectively.

### Change in annual COPD exacerbation frequency from baseline

The mean annual COPD exacerbation frequency (measured as number of episodes per year) in the past 12 months before recruitment were 0.25 ± 0.73 in the non-COVID group, 0.30 ± 0.91 in the mild-moderate COVID-19 group and 1.00 ± 1.11 in the severe COVID group. The mean annual COPD exacerbation frequency in the last year of follow-up were 0.36 ± 0.90 in the non-COVID group, 0.33 ± 1.10 in the mild-moderate COVID-19 group and 0.81 ± 1.22 in the severe COVID group.

**Figure 3:**
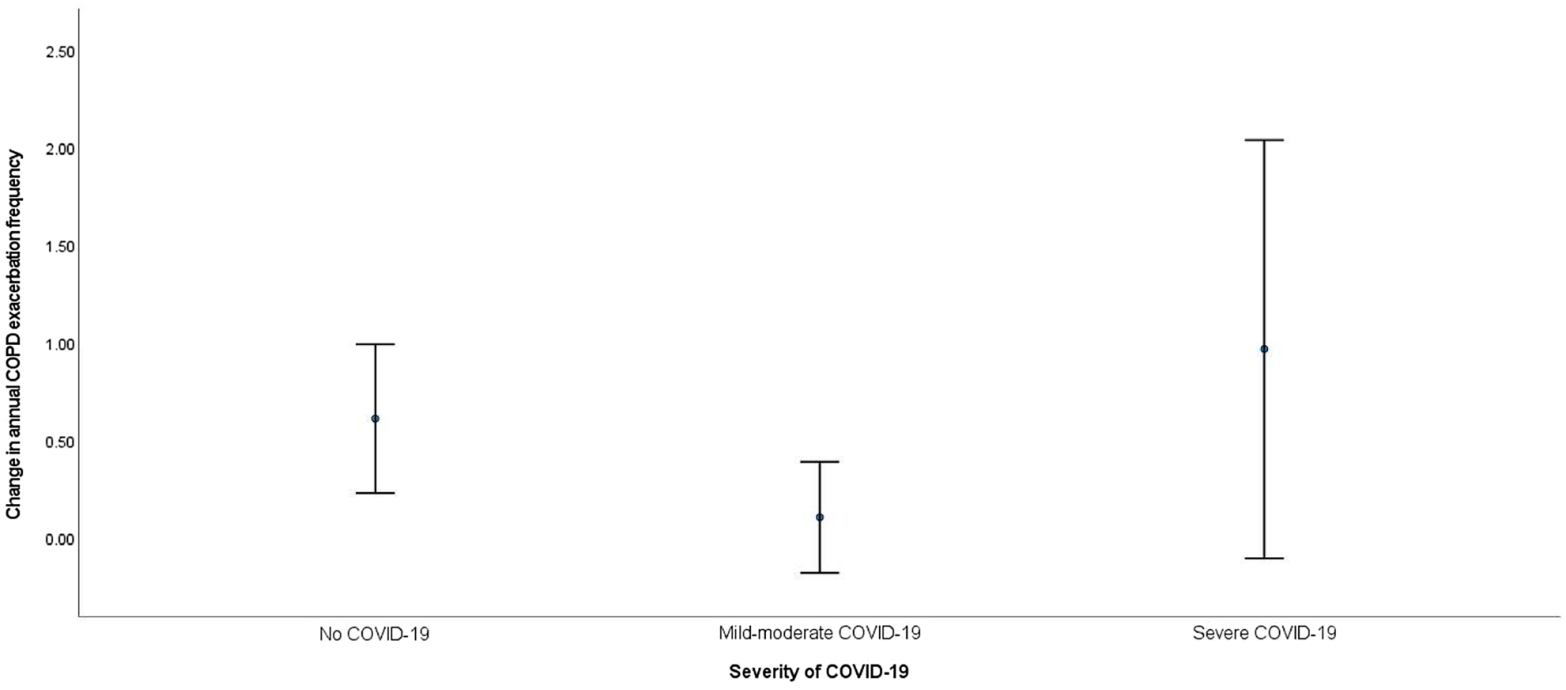
Mean change in annual COPD exacerbation frequency among patients without COVID-19, mild-moderate COVID-19 and severe COVID-19, from baseline to follow-up.

The mean change of annual COPD exacerbation frequency was + 0.31 ± 1.11 in the non-COVID group, + 0.05 ± 0.81 in the mild-moderate COVID-19 group and + 0.48 ± 1.46 in the severe COVID group, *p* = 0.046 in univariate analysis and 0.083 in multivariate analysis (Fig. 3). 27 (16.9%), 20 (15.2%) and 7 (18.9%) of the patients in the non-COVID-19, mild-moderate COVID-19 and severe COVID-19 groups had an increase in annual COPD exacerbation frequency from baseline to follow-up. The OR for increase in annual COPD exacerbation frequency, using the non-COVID-19 group for comparison, was 0.47 (95% CI = 0.16–1.33, *p* = 0.16) for the mild-moderate COVID-19 group and 1.34 (95% CI = 0.31–5.856, *p* = 0.70) for the severe COVID-19 group.

### Change in annual severe COPD exacerbation frequency from baseline

The mean annual severe COPD exacerbation frequency (measured as number of episodes per year) in the past 12 months before recruitment were 0.10 ± 0.59 in the non-COVID group, 0.14 ± 0.51 in the mild-moderate COVID-19 group and 0.19 ± 0.46 in the severe COVID group. The mean annual severe COPD exacerbation frequency in the last year of follow-up were 0.25 ± 0.64 in the non-COVID group, 0.21 ± 62 in the mild-moderate COVID-19 group and 0.74 ± 0.86 in the severe COVID group.

The mean change of annual COPD exacerbation frequency was + 0.13 ± 0.76 in the non-COVID group, + 0.07 ± 0.75 in the mild-moderate COVID-19 group and + 0.55 ± 1.01 in the severe COVID group, *p* = 0.009 in univariate analysis and 0.025 in multivariate analysis. The OR for increase in annual severe COPD exacerbation frequency, using the non-COVID-19 group for comparison, were 0.49 (95% CI = 0.28–0.86, *p* = 0.012) for the mild-moderate COVID-19 group and 4.82 (95% CI = 2.24–10.38, *p* < 0.001) for the severe COVID-19 group. The aOR were 0.51 (95% CI = 0.23–1.11, *p* = 0.09) for the mild-moderate COVID-19 group and 4.73 (95% CI = 1.55–14.41, *p* = 0.006) for the severe COVID-19 group.

### Non-COVID-19 respiratory mortality

**Figure 4:**
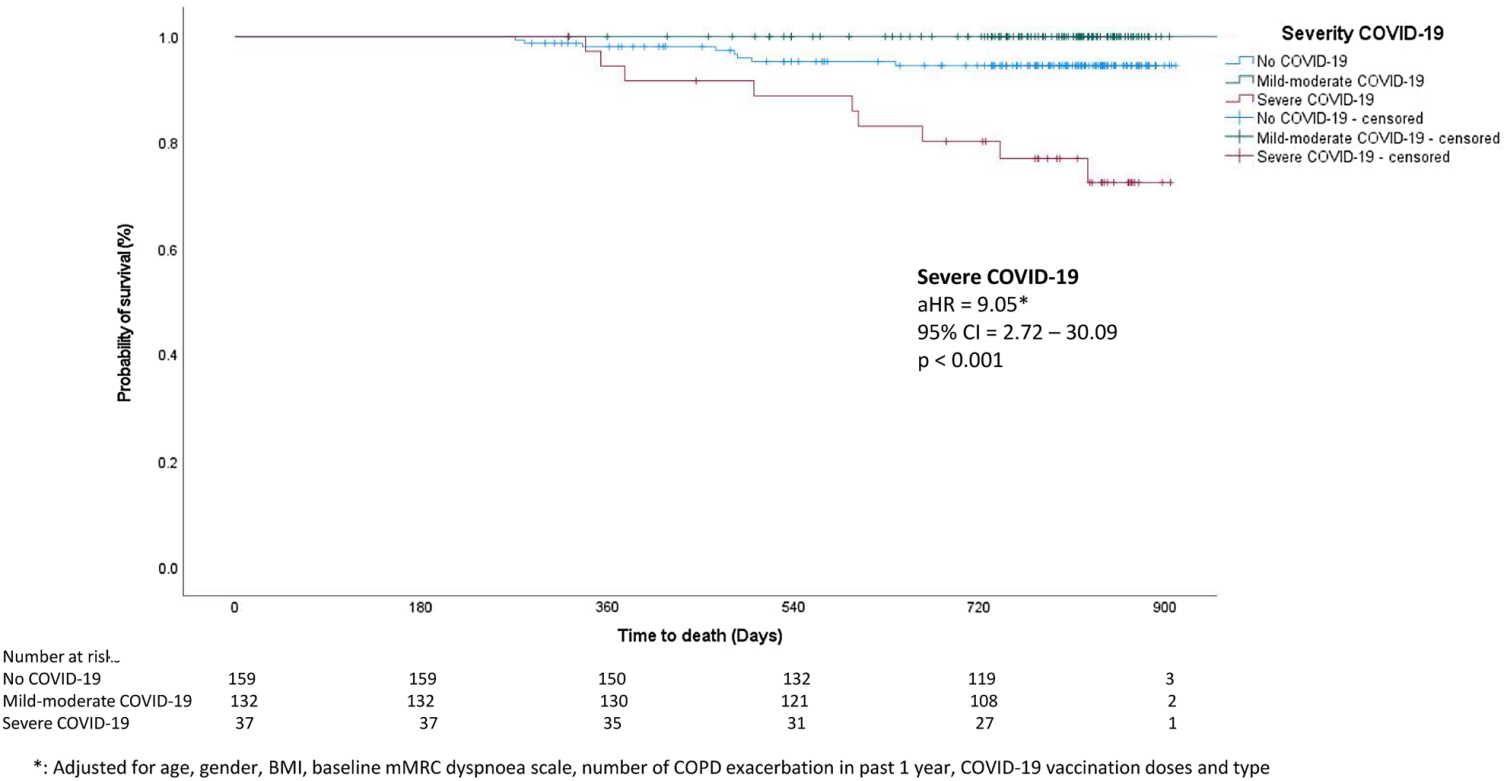
Survival analysis for non-COVID-19 respiratory mortality among patients without COVID-19, mild-moderate COVID-19 and severe COVID-19.

Among the 44 patients who died in the follow-up period, the 17 non-COVID-19 respiratory mortality comprised 8 (5.0%) in the non-COVID-19 group, 0 (0%) in the mild-moderate COVID-19 and 9 (34.3%) in the severe COVID-19 group. 22, 3 and 2 patients died of other causes were censored. There was significant increased non-COVID-19 respiratory mortality for patients in the severe COVID-19 group, comparing with the non-COVID-19 group, with hazard ratio (HR) of 4.73 (95% CI = 1.82–12.27, *p* < 0.001) and adjusted HR (aHR) of 11.25 (95% CI = 2.98–42.45, *p* < 0.001) (Fig. 4).

#### Sensitivity analysis

Sensitivity analysis was conducted among patients who had at least 2 doses of COVID-19 vaccines 14 days or more before an episode of COVID-19 infection. There were 261 patients included in the sensitivity analysis.

### Change in mMRC dyspnea scale from baseline to the last follow-up

The mean mMRC scores at baseline assessment were 1.65 ± 0.91 in the non-COVID-19 group, 1.55 ± 0.87 in the mild-moderate COVID-19 group and 1.75 ± 1.07 in the severe COVID-19 group. The mean mMRC scores on follow-up were 1.75 ± 0.97 in the non-COVID-19 group, 1.81 ± 0.92 in the mild-moderate COVID-19 group and 2.17 ± 1.03 in the severe COVID-19 group.

The mean change of mMRC dyspnea scale were + 0.09 ± 0.41 in the non-COVID-19 group, 0.23 ± 0.51 in the mild-moderate COVID-19 group and + 0.39 ± 0.50 in the severe COVID-19 group, *p* = 0.005 in univariate analysis and 0.003 in multivariate analysis. 12/134 (9.0%), 27/103 (26.2%) and 9/24 (37.5%) of the patients in the non- COVID-19, mild-moderate COVID-19 and severe COVID-19 groups had worsening of mMRC dyspnoea scale by an increased score of 1 from baseline to the last follow-up. The OR for increase in mMRC dyspnoea scale, using the non-COVID-19 group for comparison, was 3.39 (95% CI = 1.62–7.12, *p* < 0.001) for the mild-moderate COVID-19 group and 5.89 (95% CI = 2.11–16.47, *p* < 0.001) for the severe COVID-19 group, with the aOR 4.15 (95% CI = 1.66–10.34, *p* = 0.002) and 7.32 (95% CI = 2..11–25.42, *p* = 0.002) respectively.

### Change in annual COPD exacerbation frequency from baseline

The mean annual COPD exacerbation frequency in the past 12 months before recruitment were 0.25 ± 0.77 in the non-COVID-19 group, 0.22 ± 0.63 in the mild-moderate COVID-19 group and 1.13 ± 1.19 in the severe COVID-19 group. The mean annual COPD exacerbation frequency in the last year of follow-up were 0.30 ± 1.08 in the non-COVID-19 group, 0.2 ± 0.88 in the mild-moderate COVID-19 group and 0.61 ± 1.09 in the severe COVID-19 group.

The mean change of annual COPD exacerbation frequency was + 0.30 ± 1.08 in the non-COVID-19 group, + 0.02 ± 0.88 in the mild-moderate COVID-19 group and + 0.61 ± 1.67 in the severe COVID-19 group, *p* = 0.046 in univariate analysis and 0.52 in multivariate analysis.

24 (17.9%), 16 (15.5%) and 6 (25.0%) of the patients in the non-COVID-19, mild-moderate COVID-19 and severe COVID-19 groups had an increase in annual COPD exacerbation frequency from baseline to follow-up. The OR for increased annual COPD exacerbation frequency, using the non-COVID-19 group for comparison, was 0.48 (95% CI = 0.15–1.61, *p* = 0.24) for the mild-moderate COVID-19 group and 1.66 (95% CI = 0.33–8.53, *p* = 0.54) for the severe COVID-19 group.

### Change in annual severe COPD exacerbation frequency from baseline

The mean annual severe COPD exacerbation frequency (measured as number of episodes per year) in the past 12 months before recruitment were 0.11 ± 0.63 in the non-COVID group, 0.11 ± 0.46 in the mild-moderate COVID-19 group and 0.25 ± 0.53 in the severe COVID group. The mean annual severe COPD exacerbation frequency in the last year of follow-up were 0.25 ± 0.66 in the non-COVID group, 0.17 ± 0.55 in the mild-moderate COVID-19 group and 0.76 ± 0.88 in the severe COVID group.

The mean change of annual COPD exacerbation frequency was + 0.13 ± 0.78 in the non-COVID group, + 0.06 ± 0.65 in the mild-moderate COVID-19 group and + 0.50 ± 1.08 in the severe COVID group, *p* = 0.049 in univariate analysis and 0.041 in multivariate analysis. The OR for increase in annual severe COPD exacerbation frequency, using the non-COVID-19 group for comparison, were 0.66 (95% CI = 0.34–1.27, *p* = 0.21) for the mild-moderate COVID-19 group and 5.54 (95% CI = 2.21–13.88, *p* < 0.001) for the severe COVID-19 group. The aOR were 0.65 (95% CI = 0.27–1.56, *p* = 0.33) for the mild-moderate COVID-19 group and 5.62 (95% CI = 1.51–20.86, *p* = 0.01) for the severe COVID-19 group.

### Non-COVID-19 respiratory mortality

Among the 21 patients who died in the follow-up period, the 7 non-COVID-19 respiratory mortality comprised 4 (3.0%) in the non-COVID-19 group, 0 (0%) in the mild-moderate COVID-19 and 3 (12.5%) in the severe COVID-19 group. There was a significant increased non-COVID-19 respiratory mortality for patients in the severe COVID-19 group, comparing with the non-COVID-19 group, with HR 3.94 (95% CI = 0.88–17.60, *p* = 0.07) and aHR of 5.25 (95% CI = 0.74–37.28, *p* = 0.10).

## Discussion

Our study suggested that there was worsening COPD control after recovery from COVID-19 regardless of severity. The mMRC dyspnea scale increased in both mild-moderate and severe COVID-19 groups. Patients in the severe COVID-19 group also had increased risks of having more severe exacerbations and non-COVID-19 respiratory mortality (COPD exacerbation and pneumonia) after recovery. The results suggest that the delayed impact of COVID-19 on other respiratory diseases such as asthma is also present in COPD.

From the results of our study, we believe there is imminent need to arrange early follow up with appropriate actions among patients who had COPD and recent COVID-19 infection, regardless of the severity of COVID-19, as the worsening of COPD was seen across patients with mild to severe COVID-19. Patients with COPD who recovered from COVID-19 of all severity would have worsening dyspnea as measured by mMRC dyspnea scale. They will need timely assessment to consider escalation of COPD pharmacotherapy to relieve their dyspnea, which was shown to be worsened after COVID-19. Pulmonary rehabilitation should also be considered in these patients^16–18^. While patients with mild to moderate COVID-19 would have symptomatic deterioration, those with severe COVID-19 also had increase in future risks of severe exacerbation and mortality. For patients who recovered from severe COVID-19, they would need more aggressive treatment to prevent future COPD exacerbation and pneumonia, as they are at increased risks for developing severe exacerbation and also non-COVID-19 respiratory mortality due to COPD exacerbation and pneumonia after recovery from severe COVID-19. Pharmacotherapy to prevent COPD exacerbation cannot be over-emphasized. Other preventive therapy such as vaccination to prevent pneumonia caused by other micro-organisms is another important strategy to be employed^19^.

Our groups previously suggested that patients with asthma, upon recovery from mild-to-moderate COVID-19, had worsening of asthma control^8^. This phenomenon was also observed among patients with COPD, as they also had worsening symptoms as measured by mMRC dyspnea scale. And among patients with COPD, those with severe COVID-19 also had increased risks of developing future severe exacerbation and non-COVID-19 respiratory mortality. This observation can be explained by the nature of COPD. While asthma is a largely controllable disease with inhalers, biologics and bronchial thermoplasty, COPD is characterized by irreversible airflow obstruction. Patients will have gradual deterioration of lung function^20^ interposed by exacerbation^21^. Mortality from respiratory causes is almost an inevitable event. Severe COVID-19 could lead to major damages in pulmonary structure and mechanics, which may lead to subsequent fatal events such as severe COPD exacerbation and pneumonia^22^. The results from this study are alarming as the mortality from non-COVID-19 respiratory cause even exceeds that from COVID-19 itself in our cohort. This delayed fatal consequence should not be overlooked.

One important observation in this study is that there were more patients who completed COVID-19 vaccines in the non-COVID group, than the mild to moderate COVID and severe COVID-19 groups. The efficacy of COVID-19 vaccines in preventing severe COVID-19 in patients with COPD have been demonstrated in prior studies^23,24^. By preventing the development of severe COVID-19, these vaccines may be able to prevent the sequalae of severe COIVD-19 as observed in this study, including subsequent severe COPD exacerbation and mortality. Hence, the importance of completion of COVID-19 vaccination for patients with COPD should be encouraged, as we did for seasonal influenza and pneumococcal vaccines, which are within the Global Initiative for Chronic Obstructive Lung Disease recommendations^14^. The benefits from influenza and pneumococcal vaccines in patients with chronic respiratory diseases have been well demonstrated^25–31^ and the similar benefits from COVID-19 vaccines should not be forgotten.

Another point to note is the possible overuse of ICS in some of the patients in this cohort. Almost half of the patients in this cohort were on ICS, and more in the severe COVID-19 group was on ICS than the mild to moderate and non-COVID-19 groups. This could be related to the higher exacerbation number in the past 12 months in the severe COVID-19 group, leading to this possible overuse of ICS in these patients, which might increase their risks of subsequent pneumonia^32,33^. We adjusted the use of ICS and other COPD treatments in the multi-variate analysis and showed consistent results, suggesting the effect of severity of COVID-19 to be an independent predictor of the outcomes, including non-COVID respiratory mortality. However, such phenomenon still rings a bell, and it is important to review the COPD treatment records of these patients to avoid unmercenary prescription of ICS which does carry risks.

### Limitations

There are a few limitations in our study. Firstly, this study involved exclusively Chinese patients. This might affect the generalizability of the study findings in other ethnic groups. Yet, the etiology, pathophysiology and clinical features of COPD and COVID-19 are largely similar across different ethnic groups. The definition of severity of COPD and COVID-19 in our study is also consistent with the international consensus. Secondly, the patients were diagnosed with COVID-19 by RAT or PCR and therefore the measurement of viral load at the time of infection was not possible. The severity of disease and subsequent improvement were assessed based on clinical status. Thirdly, computed tomography was not available in the majority of the patients for assessment of post-COVID sequalae such as organizing pneumonia or fibrosis, which could also account for worsening of symptoms by mMRC. From chest-radiograph and clinical records, none of the patients within this cohort was diagnosed to have post-COVID organizing pneumonia or fibrosis, yet computed tomography shall be the gold standard to diagnose these conditions.

## Conclusion

After recovery from COVID-19, worsening of COPD control from worsening of dyspnea, increase in severe exacerbation frequency to non-COVID-19 respiratory mortality (COPD exacerbation and pneumonia) was observed among patients with severe COVID-19. Mild to moderate COVID-19 was also associated with symptomatic deterioration.

### Supplementary Information

Supplementary Table 1.

## Data Availability

All data generated or analysed during this study are included in this published article. The data is available from author WC Kwok upon reasonable request.

## Abbreviations

ACT: Asthma control test
COPD: Chronic obstructive pulmonary disease
COVID-19: Coronavirus disease 2019
ePR: Electronic patient record
FEV1: Forced expiratory volume in one second
FVC: Forced vital capacity
GH: Grantham Hospital
HA: Hospital Authority
ICS: Inhaled corticosteroids
LABA: Long-acting β2-agonists
LAMA: Long-acting muscarinic antagonists
mMRC: Modified Medical Research Council
QMH: Queen Mary Hospital
RAT: Rapid antigen test
RT-PCR: Reverse transcription–polymerase chain reaction
