# Supplementary material for: Outcomes among patients with chronic obstructive pulmonary disease after recovery from COVID-19 infection of different severity
Source: Sci Rep. 2024 Jun 16;14:13881. doi: 10.1038/s41598-024-64670-9 (PMC11180653; doi:10.1038/s41598-024-64670-9)
Supplement: Supplementary file 1 — Supplementary Table 1. [file 41598_2024_64670_MOESM1_ESM.docx]

**Supplementary Table 1** COVID-19 vaccination status of COPD patients according to COVID-19 infection status

|  | **Non-COVID-19**  **(n = 159)** | **Mild-to-moderate COVID-19**  **(n = 132)** | **Severe COVID-19**  **(n = 37)** | **Whole cohort**  **(n = 328)** | **p-values^** |
| --- | --- | --- | --- | --- | --- |
| Completion of COVID-19 vaccination (More than 2 doses) | 134 (84.3%) | 103 (78.0%) | 24 (64.8%) | 261(79.6%) | 0.07 |
| BNT162b2 (mRNA vaccine) | 50 (31.4%) | 35 (26.5%) | 4 (10.8%) | 89 (27.1%) |  |
| CoronaVac (inactivated whole virus vaccine) | 69 (43.4%) | 63 (47.7%) | 19 (51.4%) | 151 (46.0%) |  |
| CoronaVac (inactivated whole virus vaccine) followed by BNT162b2 (mRNA vaccine) | 15 (9.4%) | 4 (3.0%) | 1 (2.7%) | 20 (6.1%) |  |

^Between Non-COVID-19, mild-moderate COVID-19 and severe COVID-19 subgroups

*: statistically significant
